# Cu*Mn*OS Nanoflowers with Different Cu^+^/Cu^2+^ Ratios for the CO_2_-to-CH_3_OH and the CH_3_OH-to-H_2_ Redox Reactions

**DOI:** 10.1038/srep41194

**Published:** 2017-01-24

**Authors:** Xiaoyun Chen, Hairus Abdullah, Dong-Hau Kuo

**Affiliations:** 1 Department of Materials Science and Engineering, National Taiwan University of Science and Technology, Taipei 10607, Taiwan; 2 College of Material Engineering, Fujian Agriculture & Forestry University, Fuzhou 350002, China

## Abstract

A conservative CO_2_-Methanol (CH_3_OH) regeneration cycle, to capture and reutilize the greenhouse gas of CO_2_ by aqueous hydrogenation for industry-useful CH_3_OH and to convert aqueous CH_3_OH solution by dehydrogenation for the clean energy of hydrogen (H_2_), is demonstrated at normal temperature and pressure (NTP) with two kinds of Cu*Mn*OS nanoflower catalysts. The [Cu^+^]-high Cu*Mn*OS led to a CH_3_OH yield of 21.1 mmol·g^−1^catal.·h^−1^ in the Cu*Mn*OS-CO_2_-H_2_O system and the other [Cu^+^]-low one had a H_2_ yield of 7.65 mmol·g^−1^catal.·h^−1^ in the Cu*Mn*OS-CH_3_OH-H_2_O system. The successful redox reactions at NTP rely on active lattice oxygen of Cu*Mn*OS catalysts and its charge (hole or electron) transfer ability between Cu^+^ and Cu^2+^. The CO_2_-hydrogenated CH_3_OH in aqueous solution is not only a fuel but also an ideal liquid hydrogen storage system for transportation application.

Clean and renewable energies such as solar, wind, hydraulic, hydrogen, biomass energies can partially substitute for fossil fuels for the sakes of the depletion in oil resources and the emissions in greenhouse gas. The methanol economy advocated by Nobel prize winner George A. Olah in 1990 s has been promising as CH_3_OH is not only a good hydrogen liquid carrier but also a key industrial chemical feedstock^1^,^2^. The success in methanol economy is related to the reversible CO_2_ hydrogenation-CH_3_OH dehydrogenation or the CO_2_-CH_3_OH cycle. In this way, the underground fossil carbon can be conservatively exploited.

CO_2_ hydrogenation by the thermochemical conversion of the mixture of CO/CO_2_/H_2_ (termed “syngas”) for CH_3_OH operates at high temperature of ~250 °C and high pressures of ~50 bar over copper-based heterogeneous catalysts in an industrial production scale^3^,^4^,^5^. Studt *et al*. discovered a stable Ni-Ga alloy catalyst that reduced CO_2_ to methanol at ambient pressure^6^. As CO_2_ conversion to methanol is a thermodynamically exergonic process, the Ni-Ga catalyzed conversion of CO_2_ to methanol needs to be operated at and above 200 °C. To process at a mild condition, the homogeneous catalysis mainly with the use of ruthenium- and iridium-based complexes for CO_2_ hydrogenation is being developed below 145 °C under a high pressure of 60 bars^7^,^8^,^9^. Photocatalysis for CO_2_ conversion to methanol in water under high power lamp with sulfide/sulfate reagents is still an active research area^10^,^11^,^12^. Liu *et al*. demonstrated the good performance with a Cu/CeO_2_/doped graphene catalyst, but they needed the 250 W lamp illumination and Na_2_SiO_3_ reagent.

CH_3_OH dehydrogenation for hydrogen (H_2_) currently perform through steam reforming over the heterogeneous CuO/ZnO/Al_2_O_3_ catalyst at 200–300 °C^13^,^14^,^15^. The lower reaction temperature of 65–150 °C has been achieved with the active homogeneous ruthenium-complex catalysts, but additional reagents are needed^4^,^16^,^17^. Photocatalysis for aqueous alcohol conversion with a large amount of hydrogen has been achieved under high power lamp, Pt, CdS, or sulfate reagent^18^,^19^,^20^. A low operation temperature for alcohol dehydrogenation can be key factor to make CH_3_OH suitable as energy liquid carrier for portable and vehicle applications.

High temperature and pressure conversion reaction, high illumination intensity, external electrical energy input, and complex reagents have been the major approaches to make parts of the CO_2_-CH_3_OH cycle feasible for effective energy and environment managements. Therefore, the energy supplies from the perennial sources such as sun, wind, and geothermal to execute the reactions for fuels and chemicals have been widely pursued. Recently, metallic AgPd hollow sphere anchored on graphene demonstrated the dehydrogenation of formic acid for H_2_ at room temperature^21^. It is encouraging that the catalytic reaction with AgPd for H_2_ production without thermal, electrical, and photo energies is not impossible to occur. Here, we demonstrate two kinds of inorganic Cu*Mn*OS catalysts with low cost: the [Cu^+^]-high Cu*Mn*OS acts as catalyst to accelerate the reduction reaction of the CO_2_ hydrogenation to methanol and the [Cu^+^]-low Cu*Mn*OS to speed up the oxidation reaction of the methanol dehydrogenation into hydrogen and carbon dioxide, to complete the conservative CO_2_-CH_3_OH cycle at normal temperature and pressure without additional reagents.

## Methods

### Synthesis of CuMnOS

To prepare Cu*Mn*OS powder, 1.5 g thioacetamide (CH_3_CSNH_2_) was added into a 500 ml solution with cupric nitrate (Cu(NO_3_)_2_·2.5H_2_O) and manganese (II) chloride (MnCl_2_) in the weight ratio of 1: 1, followed by 30 min stirring. Then the mixture solution was steadily heated to 95 °C and 0.0, 0.1, 0.2, 0.3 and 0.4 ml hydrazine (N_2_H_4_) were added to prepare the powders at different redox conditions. After stirring for 2 h, the precipitates were collected after centrifugation and washing procedures. The precipitates were dried in oven at 80 °C for 24 h. The obtained catalysts were labeled as Cu*Mn*OS-0, Cu*Mn*OS-1, Cu*Mn*OS-2, Cu*Mn*OS-3, and Cu*Mn*OS-4, depending upon their N_2_H_4_ content. For comparative purpose, the CuOS was prepared at the same procedure without adding MnCl_2_.

### Characterization of CuMnOS

The photoelectron spectrometry (XPS) was proceeded with VG Scientific ESCALAB 250 XPS under the Al Kα X-rays (*hv* = 1486.6 eV) radiation and calibrated with carbon C1*s (E*a = 284.62 eV). The X-ray powder diffraction (XRD) study was conducted on Bruker D2 phaser X-ray diffractometry at 10 kV using Cu Kα radiation at a scanning step size of 0.05° and with residence time of 0.5 min. SEM images were obtained from JSM-7610F field-emission scanning electron microscope (FE-SEM) operated at an accelerating voltage of 15 kV. A Tecnai F20 G2 instrument was used to obtain the TEM images and microstructural information. To obtain the specific surface area (*S*_BET_), N_2_ adsorption-desorption experiments were performed on Micromeritics ASAP 2020 porosity and specific surface area analyzer after the sample degassed at 150 °C for 2 h. UV-Vis DRS was evaluated on a JASCD V-670 UV-Vis spectrophotometer with an integrating sphere of 60 mm and BaSO_4_ as a reference material. Photoluminescence (PL) emission spectrum was measured at room temperature on JASCD FB-8500 fluorescence spectrophotometer with a laser beam at 330 nm emission wavelength.

### Reduction/hydrogenation reactions

#### Reduction of Cr(VI) by the catalytic reduction reaction of CuMnOS

To execute the reduction of Cr(VI), the 50 mg catalyst was added into the reactor filled with 100 mL Cr(VI) solution of 50 mg/L. The reactor also was wrapped with aluminum foil. After reaction for 2 min, approximately 8 mL sample was taken out and passed through a 0.45 μm membrane filter syringe to immediately separate catalysts from the solution. The diphenycarbazide (DPC) colorimetric method^22^ with JASCD V-670 spectrophotometer and the ion chromatography (IC) method with Thermo ICS-5000 spectrophotometer were used to determine the Cr(VI) concentration in filtrate. To evaluate the reusability, the catalysts after the first run were re-used for the second run at the same condition after re-filling with a fresh Cr(VI) solution without being washed. For this reusability purpose, a larger amount of 50 mg catalyst was used to avoid the larger deviation caused by the weight loss.

#### Reduction conversion of CO_2_ by aqueous hydrogenation with CuMnOS catalyst

Reduction conversion of CO_2_ to CH_3_OH with Cu*Mn*OS was carried out in a home-made and jacketed quartz reactor wrapped by aluminum foil. For each run, the 0.1 g catalyst was added into the reactor with 70 mL distilled water, then CO_2_ gas, released from NaHCO_3_ solution by controlling the addition of dilute HNO_3_ aqueous solution, was passed into the reactor under the ambient laboratory condition. The whole procedure lasted for 18 h. The products were collected and analyzed by GC with flame ionization detector (FID).

### Oxidation/dehydrogenation reactions

#### Degradation of methylene blue by dye oxidation reaction with CuMnOS catalyst

To proceed the MB degradation experiments, the 25 mg catalyst was added into the reactor filled with 100 mL MB solution of 10 mg/L. The reactor was wrapped with aluminum foil to exclude the effects of UV and visible light irradiations. The 3 mL sample was taken out from the reactor every 5 min, followed by instant centrifugation in 1 min. The supernatant absorbance was measured with a JASCD V-670 UV-Vis spectrophotometer for peak located at 663 nm. Their concentration was calculated based on the Lambert-Beer law. To evaluate the reusability, the catalysts after the first run were re-used for the second run at the same condition after re-filling with a fresh MB solution without being washed.

#### Hydrogen generation by catalytic dehydrogenation (aqueous oxidation) with CuMnOS

Hydrogen generation was conducted in a home-made and jacketed quartz reactor equipped with the input and output valves to control the gas flow. To exclude the visible light irradiation, the reactor was wrapped by aluminum foil. One Cu*Mn*OS sample was compared by exposure under the 150 W Halogen lamp illumination. The input valve was connected to a gas tank of 99.99% Ar and the output one to a well-callibrated gas chromatography (GC) with thermal conduction detector (TCD) system. The hydrogen evolution experiment was carried out with the well-dispersed 225 mg catalyst in 450 mL pure ethanol (C_2_H_5_OH), water, ethanoic acid, or the methanol (CH_3_OH), ethanol, or ethanoic acid aqueous solution (20% v/v). The gas sampling was taken for each time interval of 20 min. Gas sampling was conducted by flowing Ar gas through the reactor to GC-TCD system for several minutes. A hydrogen calibration line was built to quantitatively measure the H_2_ generation rate.

## Results

### XPS analysis

**Figure 1:**
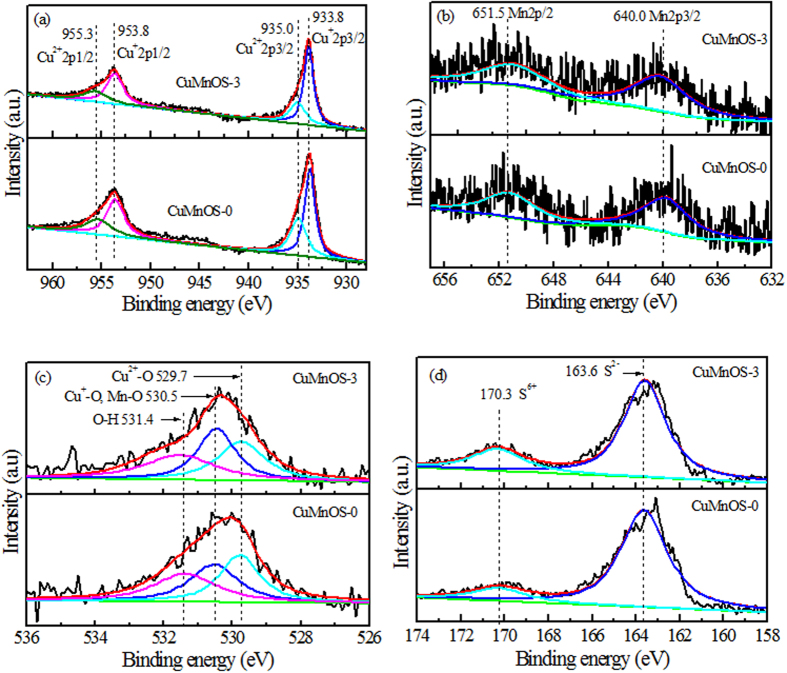
High resolution (**a**) Cu2*p*, (**b**) Mn2*p*, (**c**) O1*s*, and (**d**) S2*p* XPS spectra of Cu*Mn*OS-0 and Cu*Mn*OS-3.

The compositions of Cu*Mn*OS-0 and Cu*Mn*OS-3 are listed in Table 1 after the XPS analyses. The Cu, Mn, O, and S contents are close for the two kinds of catalysts prepared at different N_2_H_4_ contents. The substitutional Mn has a Mn/(Mn + Cu) molar ratio of ~0.038, a much lower content than the Cu content. Figure 1a shows the high resolution Cu2*p* XPS spectra of Cu*Mn*OS-0 and Cu*Mn*OS-3. The asymmetric Cu2*p* peaks were contributed to the different chemical states of Cu in Cu*Mn*OS. The peak separation of 20.0 eV between Cu2*p*3/2 and Cu2*p*1/2 located at 933.8 eV and 953.8 eV, respectively, indicates that copper is for the monovalent Cu^+ ^23. The peaks of 2*p*3/2 and 2*p*1/2 located at 935.0 eV and 955.3 eV, respectively, were attributed to the spin-orbit splitting of the bivalent Cu^2+ ^24,25. According to the quantitative analysis by integrating the peak area, both of catalysts are richer in Cu^+^ than Cu^2+^ and the Cu^+^/Cu^2+^ molar ratios were calculated to be 1.49 for Cu*Mn*OS-0 and 2.39 for Cu*Mn*OS-3. With increasing the reducing N_2_H_4_ content, the Cu^+^/Cu^2+^ molar ratio increased or the Cu^2+^ → Cu^+^ transition was accelerated. Cu*Mn*OS-0 without adding N_2_H_4_ has a lower Cu^+^ content and it is labeled as [Cu^+^]-low Cu*Mn*OS. Cu*Mn*OS-3 had a higher Cu^+^ content after adding N_2_H_4_ during the preparation stage and it is labeled as [Cu^+^]-high Cu*Mn*OS.

Figure 1b shows the high resolution Mn2*p* XPS *spectra* of Cu*Mn*OS-0 and Cu*Mn*OS-3. The peak separation of 11.5 eV between Mn2*p*3/2 and Mn2*p*1/2 located at 640.0 eV and 651.5 eV, respectively, indicates that copper is for the bivalent Mn^2+ ^26,27. Figure 1c shows the high resolution O1*s* XPS spectra of Cu*Mn*OS. The asymmetric O1*s* peak was convoluted into three kinds of peaks at 531.4 eV contributing from the hydroxyl oxygen^28^, at 530.5 eV from the Mn-O and monovalent Cu^+^ -O^29^,^30^, and 529.7 eV from the bivalent Cu^2+^ -O^31^. Figure 1d shows the high resolution S2*p* XPS spectra of Cu*Mn*OS. The S2*p* peaks at 163.6 eV belongs to the S^2- ^32,33 and at 170.3 eV to S^6+ ^34. With increasing the reducing N_2_H_4_ content, the S^6+^ content in Cu*Mn*OS, for the sake of charge neutrality, increases to compensate the loss in the positive charge due to the Cu^2+^ → Cu^+^ transition. That is to say, [Cu^+^]-low Cu*Mn*OS-0 has a lower S^6+^ content and [Cu^+^]-high Cu*Mn*OS-3 a higher one. The Cu^+^ content is proportional to the S^6+^ content in Cu*Mn*OS. The lattice O^−2^/S^−2^ molar ratio, removing the contribution from the hydroxyl oxygen, is ~0.466 for both of catalysts with a slightly higher S^2-^ ratio.

### XRD analysis

**Figure 2:**
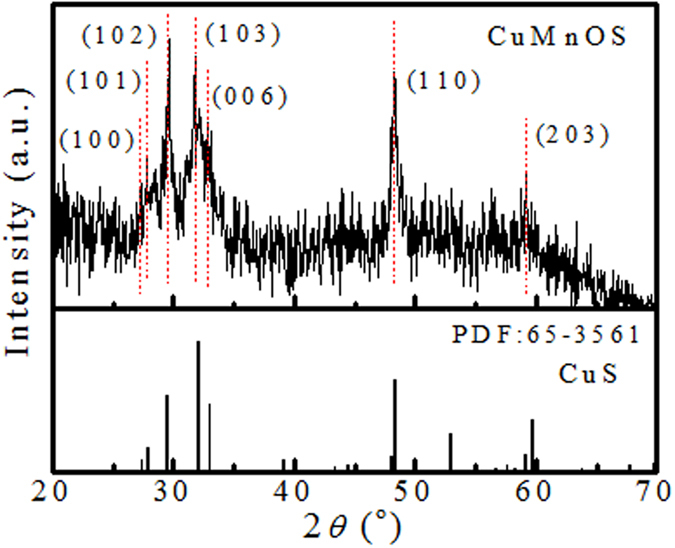


Figure 2 shows the XRD diffraction pattern of Cu*Mn*OS and the standard peaks of CuS based upon PDF # 65-3561. The diffraction peaks of Cu*Mn*OS-0 were the same as those of Cu*Mn*OS-3. XRD peak positions of Cu*Mn*OS were well matched to those of the hexagonal CuS covellite structure. The main peaks located at 27.66°, 28.36°, 29.62°, 31.81°, 32.72°, 48.23°, and 59.15° attributed to the (100), (101), (102), (103), (006), (110), and (203) crystal planes, respectively. The weak and broad Cu*Mn*OS peaks are attributed to the poor crystallization. XRD diffraction pattern did not show any second phase.

### SEM and TEM microstructural and structural analyses

**Figure 3:**
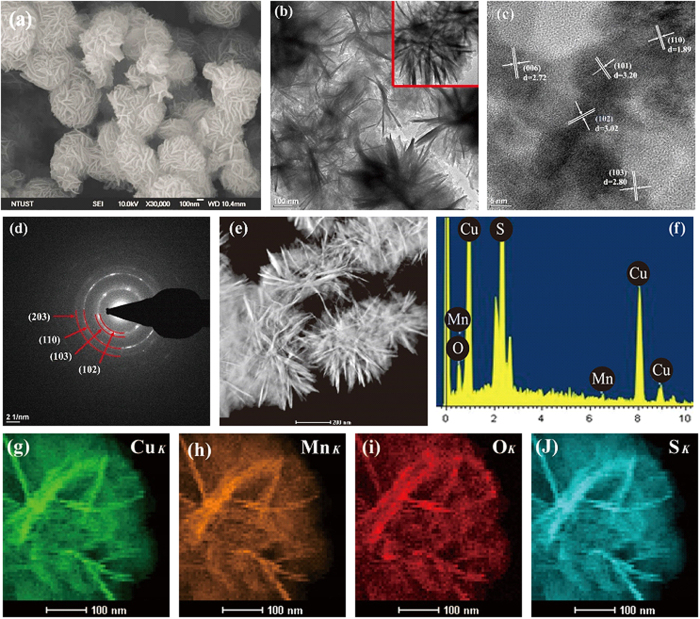
Microstructural and structural characterizations of Cu*Mn*OS-3. (**a**) FE-SEM image, (**b**) TEM image, (**c**) HR-TEM image, (**d**) SAED pattern, (**e**) HAADF-STEM image, (**f** ) FE-SEM-EDS spectrum, (**g**–**j**) EDX elemental mapping of Cu, Mn, O, and S, respectively. The inset in (**b**) is for image at higher magnification.

Figure 3a shows the FE-SEM images of Cu*Mn*OS-3. Cu*Mn*OS looks like the petal-gathered nanoflower particles with its size of 300~500 nm. Similar to Cu*Mn*OS-3 in FE-SEM image, Cu*Mn*OS-0 was not displayed. Figure 3b shows the TEM image of Cu*Mn*OS to further verify its nanoflower-like microstructure. The inset in Fig. 3b shows the image at higher magnification. Figure 3c shows the HR-TEM image of Cu*Mn*OS. Different lattice fringes belonging to different grains were observed, indicating the nature of nanoparticles. Figure 3d shows the selected area electron diffraction (SAED) pattern of Cu*Mn*OS-3. The ring patterns from the (102), (103), (110) and (203) planes explain its polycrystalline nature. The scattered ring pattern explains the solid solution nature of Cu*Mn*OS. Figure 3e gives the HAADF-STEM image, which reveals many pores with different sizes inside the nanoflower-like Cu*Mn*OS particles. Figure 3f shows the FE-SEM-EDS spectrum, which verifies that aggregates are composed of Cu, Mn, S, and O. Figure 3g–j show the HAADF-STEM-EDX elemental maps of Cu, Mn, O, and S. From these element mappings, we can confirm the composition uniformity in samples.

### UV-Vis absorption and photoluminescence

**Figure 4:**
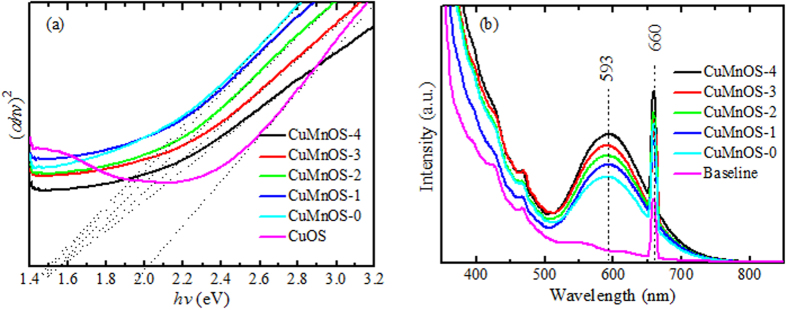
(**a**) the (α*hν*)^2^-*hν* plot from the optical absorption measurements for determining the bandgap, and (**b**) PL spectra of Cu*Mn*OS catalysts prepared at different N_2_H_4_ contents.

The optical absorption property of Cu*Mn*OS was characterized by UV-Vis absorption spectroscopy. Cu*Mn*OS had a better visible light absorbance than CuOS. From the UV-Vis spectra, the direct band gap was measured with the equation versus photon energy (*hν*)^35^:


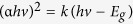


where α is the absorbance coefficient, *h* the Planck constant, *k* the absorption constant for a direct transition, *hν* the absorption energy, and *E*_g_ the band gap. Figure 4a shows the (α*hν*)^2^-*hν* curves of Cu*Mn*OS together with the comparative CuOS. The *E*_g_ values were determined to be 2.0 eV for CuOS and 1.5~1.6 eV for Cu*Mn*OS with the higher value at the higher Cu^+^ content in Cu*Mn*OS. The variation of energy band gap further indicates that Cu*Mn*OS is a bimetal oxysulfide solid solution instead of monocrystalline CuO with band gap of *E*_g_ = 1.2–1.4 eV, Cu_2_O of 2.0–2.2 eV, CuS of 2.15–2.36 eV, and Cu_2_S of 1.2–1.25 eV.

Figure 4b shows PL spectra of the Cu*Mn*OS catalysts. Under a laser beam at wavelength of 330 nm, catalysts were excited with PL spectra at about 593 nm. The peak at 660 nm is originated from the laser contribution. It is observed that the 593 nm peak intensity increases with the N_2_H_4_-adding content or the Cu^+^ content. The more Cu^+^ content in Cu*Mn*OS-3 can contribute the more defect levels to lead to the higher emission intensity.

### BET and pore size analyses

**Figure 5:**
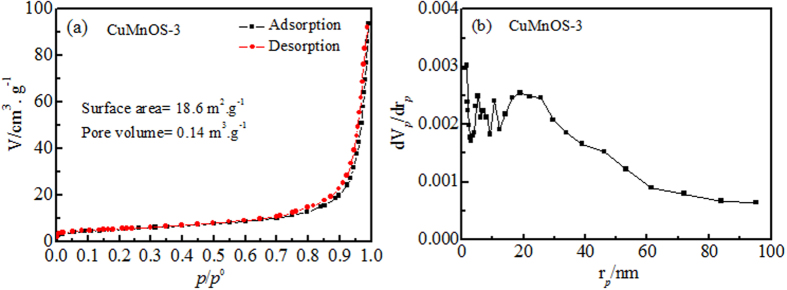
(**a**) Nitrogen adsorption-desorption isotherm and (**b**) pore size distribution curve of Cu*Mn*OS.

Figure 5a shows the N_2_ adsorption-desorption isotherm of Cu*Mn*OS, which displays the type IV isotherm with the hysteresis loop at relative pressure (*P*/*P*_0_) between 0.75 and 1.0, indicating its mesoporous feature^36^. Figure 5b shows the pore size distribution of Cu*Mn*OS. Cu*Mn*OS-0 and Cu*Mn*OS-3 had the surface area (*S*_BET_) of 20.3 and 18.6 m^2^/g, the total pore volumes of 0.151 and 0.141 cm^3^/g, and the average pore diameters of 30.5 and 30.4 nm, respectively. The large pore diameter is attributed to the aggregation of the petal-gathered nanoflower particles.

### Reduction activity of CuMnOS on Cr(VI)

Table 2 shows the reduction of Cr(VI) over Cu*Mn*OS and CuOS catalysts in the dark. The different Cu*Mn*OS catalysts performed differently in Cr(VI) reduction with the efficiency in the order: Cu*Mn*OS-4 ≈ Cu*Mn*OS-3 > Cu*Mn*OS-2 > Cu*Mn*OS-1 > Cu*Mn*OS-0 > CuOS. The Cu*Mn*OS-3 and Cu*Mn*OS-4 catalysts completed the Cr(VI) reduction in 2 min, while CuOS only completed 8.5%. As tested by IC method, the Cr(VI) in solution was confirmed to be reduced to Cr^0^ without the existence of Cr^3+^. In order to test the catalytic capability and their reusability, Cu*Mn*OS was continuously tested for three runs. After the 3^rd^ run, the Cu*Mn*OS-3 still maintained the good catalytic activity to reduce more than 97.4% of Cr(VI). The results indicate that the bimetal [Cu^+^]-high Cu*Mn*OS oxysulfide catalysts prepared with a higher N_2_H_4_ amount show excellent catalytic activity without the needs of other chemicals and photo energy. The [Cu^+^]-high Cu*Mn*OS is promising for industrial applications in Cr(VI) waste water treatment.

The experimental methods for Cr(VI) depollution include photocatalysis and absorption with high surface energy nanomaterials. As the rate constant is affected by the catalyst amount, illumination light intensity etc., the quantity of K_2_Cr_2_O_7_ amount (mg) divided by catalyst amount (mg), i.e. W_2,(K2Cr2O7)_/W_1,(catalyst)_, is used for comparison. Under the UV light, TiO_2_-CNT with a W_2_/W_1_ value of 0.013 reduced 100% Cr(VI) in 40 min^37^. Under the visible light, Fe_2_O_3_/g-C_3_N_4_ with a W_2_/W_1_ value of 0.014 reduced 100% Cr(VI) in 15 min^38^. With no light illumination, diamino pyridine-modified grapheme oxide with a W_2_/W_1_ value of 0.5 absorbed 94.5% Cr(VI) in 90 min at the help of the electrostatic force^39^. In the dark condition, our Cu*Mn*OS with a W_2_/W_1_ value of 0.1 reduced Cr(VI) in 2 min. The Cu*Mn*OS catalyst had demonstrated the excellent ability in the reduction of Cr(VI).

### Aqueous hydrogenation conversion of CO_2_

Table 3 shows the yields of CH_3_OH in conversion of CO_2_ over Cu*Mn*OS and CuOS. It is interesting to note that pure CuOS did not produce CH_3_OH. However, the aqueous hydrogenation of CO_2_ by Cu*Mn*OS to produce CH_3_OH with the yield in the order: Cu*Mn*OS-3 > Cu*Mn*OS-2 > Cu*Mn*OS-1 > Cu*Mn*OS-4 > Cu*Mn*OS-0. The CH_3_OH yield increased with the Cu^+^ content in Cu*Mn*OS but reached the highest yield of 21.1 mmol·g^−1^catal.·h^−1^ at Cu*Mn*OS-3. The [Cu^+^]-high Cu*Mn*OS favors the aqueous hydrogenation of CO_2_. In the industrial scale, thermal conversion above 200 °C had a rate above 60 mmol·g^−1^catal.·h^−1 ^40. For photo conversion, the maximal rate of 0.51 mmol·g^−1^catal.·h^−1^ was achieved by the Cu-CeO_2_ system with the 250 W Xe lamp^10^. Our production in the dark with a rate of 21.1 mmol·g^−1^catal.·h^−1^ is quite promising.

### Degradation of methylene blue by oxidation reaction

**Figure 6:**
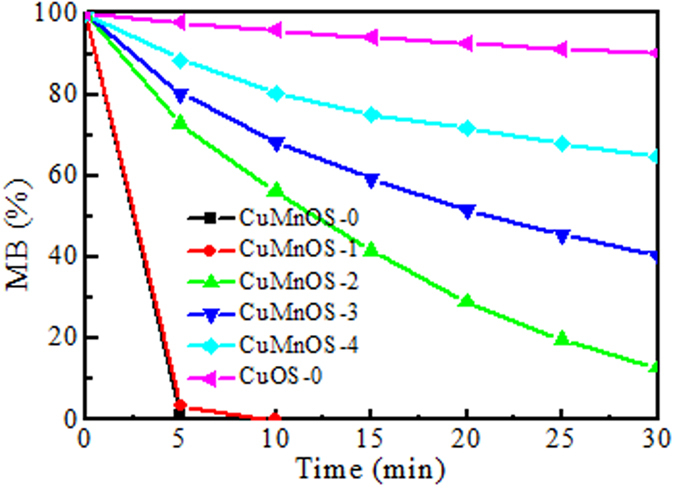


Figure 6 shows the degradation of MB over different catalysts in the dark. It is noted that N_2_H_4_ added in processing has an important effect for preparing Cu*Mn*OS on the degradation of MB with the efficiency in the order: Cu*Mn*OS-0 ≈ Cu*Mn*OS-1 > Cu*Mn*OS-2 > Cu*Mn*OS-3 > Cu*Mn*OS-4. Cu*Mn*OS-0 and Cu*Mn*OS-1 could completely degrade MB in 5 min. However, the CuOS catalyst only removed 9.9% MB in 30 min. In order to test the catalyst reusability, the supernatant of Cu*Mn*OS-0 catalyst after the first test and gravity setting was decanted and then the fresh 100 mL MB solution of 10 ppm was added for the reuse test in the dark without washing catalysts. The 2^nd^ run was also completed in 5 min. After the 3^rd^ run, the Cu*Mn*OS remained effective to degrade 95% MB in 5 min. To differentiate the dye degradation or adsorption, the wash-out ethanol solution of Cu*Mn*OS powder after the 3^rd^ run was analyzed with UV-Vis spectrophotometer at 663 nm. The disappearance of the characteristic peak confirmed there was no MB absorption on Cu*Mn*OS. A comparative experiment on activated carbon with *S*_BET_ above 1000 m^2^/g did show the characteristic peak at 663 nm for MB after the carbon powder was washed. Our Cu*Mn*OS has a too low S_BET_ value of ~20 m^2^/g for adsorption to proceed, so the MB dye here looks to be degraded by the catalytic reaction in the dark. Together with the XPS data, the [Cu^+^]-low Cu*Mn*OS dominates over [Cu^+^]-high one for the MB oxidative degradation.

There are some reports on the MB degradation in the dark. The quantity of MB amount (mg) divided by catalyst amount (mg), W_2,(MB)_/W_1,(catalyst)_, is used for evaluation. Ag-In-Ni-S nanocomposites with a W_2_/W_1_ value of 0.0022 degraded 98% MB in 12 min^41^. NiS nanoparticles with a W_2_/W_1_ value of 0.0089 degraded 100% MB in 15 min^42^. CuS caved superstructure with a W_2_/W_1_ value of 0.01 degraded 100% MB in 15 min with the help of hydrogen peroxide^43^. Our Cu*Mn*OS with a W_2_/W_1_ value of 0.04 degraded 100% MB in 5 min. The Cu*Mn*OS catalyst had demonstrated the admirable ability in the degradation of MB.

### Hydrogen production by aqueous CH_3_OH dehydrogenation

The results of hydrogen production by aqueous CH_3_OH dehydrogenation with the Cu*Mn*OS catalysts prepared at different N_2_H_4_ contents are shown in Table 4. It is interesting to mention that the catalyst in each pure H_2_O, alcohol, and organic acid did not generate hydrogen, but the aqueous solutions of alcohol and organic acid produced H_2_ at NTP in the dark. For the mixture solution of alcohol and organic acid without water, it did not work out for H_2_ generation, either. These results indicate that hydrogen generation process involves the catalytic reactions with water and alcohol, or water and organic acid. The reaction between catalyst and water is especially critical. Without the existence of water to participate, hydrogen does not produce. From Table 4, the highest H_2_ yield of 7.65 mmol·g^−1^catal.·h^−1^ in the methanol solution and 9.45 mmol·g^−1^catal.·h^−1^ in the ethanol solution, are much higher than that of CuOS at 0.270 mmol·g^−1^catal.·h^−1^. The H_2_ generation also occurred for Cu*Mn*OS-0 with the yield of 2.17 mmol·g^−1^catal.·h^−1^ in an ethanoic acid solution. Under the 150 W Halogen lamp visible illumination, the H_2_ yield degraded to 2.04 mmol·g^−1^catal.·h^−1^ in methanol solution. The ineffectiveness of photo-induced electron-hole pairs under light illumination for H_2_ production at NTP indicates dehydrogenation of aqueous CH_3_OH by Cu*Mn*OS-0 is not initiated by the electron/hole charges. After Cu*Mn*OS-0 nanoflower catalyst was annealed at 200 °C, its H_2_ yield further degraded to 1.20 mmol·g^−1^catal.·h^−1^ due to the deactivation of the catalyst activity. There is a trend of lowering the H_2_ yield at the higher Cu^+^ content for preparing Cu*Mn*OS with the higher N_2_H_4_ amount. To test catalyst reusability, Cu*Mn*OS-1 catalyst after the 24 h immersing in alcohol solution, drying, and re-filling was tested again and its H_2_ yield of 6.63 mmol·g^−1^catal.·h^−1^ was achieved.

H_2_ evolution has been studied by different routes. For the photocatalytic reactions in the water/methanol solution, Sun *et al*. with Ni_2_P/CdS nanorods obtained a record-high H_2_ production rate of 553 mmol·g^−1^catal.·h^−1^ under a filtered 300 W Xe lamp. The rate per input light power can be viewed as 1.84 mmol·g^−1^catal.·h^−1^·watt^−1 ^44. The other excellent catalyst was Sr-NaTaO_3_ with a rate of 48.9 mmol·g^−1^catal.·h^−1^ or 0.79 mmol·g^−1^catal.·h^−1^·watt^−1 ^45. Ruthenium hydride complex performed the best for the homogeneous catalysis of methanol and water into CO_2_ and H_2_O at 90 °C under the additive of KOH^7^. Without the precious metal, the H_2_ production rate is low. Our Cu*Mn*OS-1 with a rate of 9.45 mmol·g^−1^catal.·h^−1^ in the dark is encouraging.

## Discussion

The developments of the pure electron-transport catalyst for reduction/hydrogenation of CO_2_ into methanol and the pure hole-transport one for oxidation/dehydrogenation of aqueous alcohol into H_2_ without the thermal, electrical, and photo energies are our major goals. We adopt the hexavalent Cr reduction and dye degradation for screening the redox capability during our search for catalysts. Compared with the reported redox reactions for the pollutant removal, our catalytic reactions are pretty fast at NTP. To further test the redox capability with our Cu*MnO*S system, aqueous CO_2_ hydrogenation is used for testing the catalytic reduction reaction and aqueous CH_3_OH dehydrogenation for oxidation one. The first evidence for the success in the hydrogenation-dehydrogenation redox reactions is the content of the different Cu charge states. The [Cu^+^]-high Cu*Mn*OS is used and good for CO_2_ reduction, therefore it can transport electrons through the Cu^+^/Cu^2+^ charge centers for the solution/catalyst interface reaction. The [Cu^+^]-low Cu*Mn*OS is used for aqueous CH_3_OH dehydrogenation, therefore it can transport holes through the Cu^+^/Cu^2+^ charge centers.

For CH_3_OH generation from the simple Cu*Mn*OS-CO_2_-H_2_O system, the formation of proton is needed, followed by the reaction with CO_2_ for forming CH_3_OH. For H_2_ generation from the simple Cu*Mn*OS-CH_3_OH-H_2_O system at NTP, it needs any one of CH_3_OH, H_2_O, and Cu*Mn*OS added to the mixture of the other two, otherwise there is no H_2_ gas release. This observation gives a hint that a series reaction operates in this system. To logically explain the complex reactions in each of the simple systems, our catalyst has to be quite active and can react in the CO_2_-H_2_O or CH_3_OH-H_2_O solution with H_2_O existing in both situations. To make the series reaction happen and to explain the rare phenomena, the catalyst has to firstly react with H_2_O, followed by the reaction with CO_2_ in the Cu*Mn*OS-CO_2_-H_2_O system or with CH_3_OH in the Cu*Mn*OS-CH_3_OH-H_2_O one, as we had mentioned about the critical role of H_2_O. For catalyst to be active, its lattice bonding on surface needs to be weak for the interfacial exchange reactions. The degraded performance for the 200 °C-annealed Cu*Mn*OS in Table 4 is a support related to lattice bonding. Therefore, the second key factor for the success in the redox reactions is the weakened lattice oxygen at the catalyst surface to have its active lattice oxygen easily react with water for forming the oxygen vacancy and the oxidized OH^-^ on catalyst surface. The Kröger–Vink notation originally developed for ionic compounds is used here. For the oxygen vacancy (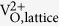) as an example, the main body of V represents for vacancy, the subscript for the host lattice site, and the superscript for the relative charge. Here we adopted the positive charge of 2+ instead of the “••” symbol in the original invention. For the active lattice oxygen, it is shown as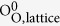. Therefore, *water* oxidation reaction is shown below in term of the Kröger–Vink notation:


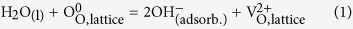


In the above Eq. 1, the mass, charge, and lattice site are required to be conservative. The active lattice oxygen on surface and the generated oxygen vacancy become the oxidant and the reducing agents, respectively. The redox reactions by oxide defects in CeO_2_ had been used for thermochemical catalytic production of solar fuels above 1000 °C^46^,^47^. Here we just perform the similar reactions in the liquid state at much lower temperature. Before discussing the CO_2_ hydrogenation and CH_3_OH dehydrogenation, a common reaction in the Cu^2+^/Cu^+^ -coexisting compounds is listed below for the consideration of the reaction reversibility:


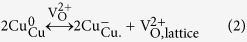


where 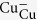 represents for the occupation of Cu^+^ on the Cu^2+^ lattice site with a relative negative charge of 1- and 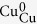 for the Cu^2+^ on the Cu^2+^ lattice site.

For the [Cu^+^]-high Cu*Mn*OS-CO_2_-H_2_O reaction system to form CH_3_OH at NTP, the reducing agent of oxygen vacancy can be oxidized by H_2_O to form the active lattice oxygen on catalyst and 2 H^+^ at the solid/liquid interface (Equation 3). The protons together with the hopping electrons between Cu^+^ and Cu^2+^ in the [Cu^+^]-high Cu*Mn*OS, shown in Eq. 4, can reduce the dissolved and adsorbed CO_2_ into CH_3_OH by the catalyst/solution interface reaction in Eq. 5. After combining Eqs. 2, 3, 4, and 5, the net equation 6 is obtained. Consistent with the data in Table 3, the mechanism explains that the increased Cu^+^ content favors the electron formation in Eq. 4 and the CH_3_OH yield in Eq. 5. Aided by 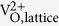 in Eq. 6, the formed 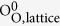 can continuously proceed Eq. 1 to have the reversible reaction and the Cu*Mn*OS catalyst has its surface kept at dynamic equilibrium between 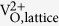 and 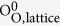.


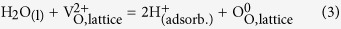



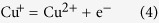



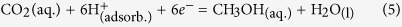



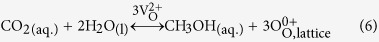


The kinetic reaction steps in Eqs. 1 and 3 demonstrate the lattice oxygen in and out at the catalyst/solution interface to hold the dynamic equilibrium and to keep Cu*Mn*OS behave as a catalyst for a long period of reaction and for repeated use without being exhausted. For the reaction to continuously run, the continuous supply of electrons for Eq. 4 is needed. The establishment of thermal equilibrium in Eq. 2 is also important to avoid the electron depletion.

For the [Cu^+^]-low Cu*Mn*OS-CH_3_OH-H_2_O reaction system to form H_2_ at NTP, the reduced oxygen vacancy reacts with H_2_O for *water* reduction to form H_2_, two electrical holes (2 h^+^), and active lattice oxygen, as shown in Eq. 7. The hydroxyl group from Eq. 1 together with the hopping holes between Cu^+^ and Cu^2+^ in the [Cu^+^]-low Cu*Mn*OS, shown in Eq. 8, can oxidize CH_3_OH into 5/2H_2_ and CO_2_, as shown in Eq. 9. After combining Eqs. 1, 7, and 9, the net equation 10 is obtained. With this proposed mechanism, it can explain the fact that the aqueous methanol dehydrogenation cannot occur without the initiation of the water oxidation reaction in Eq. 1. It also explains the increased Cu^+^ content unfavors the hole formation in Eq. 8 and the H_2_ yield in Eq. 9, as supported by the data in Table 4.


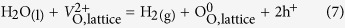



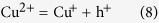



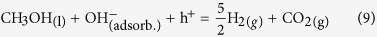



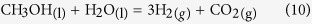


Thermodynamic consideration for the CO_2_ hydrogenation is evaluated to support the feasibility of the reaction in Eq. 6, which can be divided into Eqs. 11 and 12:


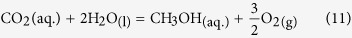



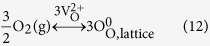


**Figure 7:**
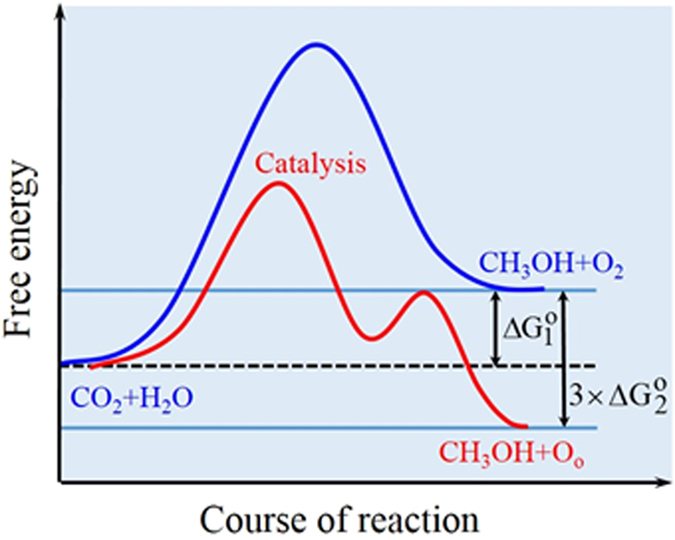


The standard Gibbs free energies of formation of aqueous methanol, O_2(g)_, CO_2(aq.)_, and H_2_O_(l)_ are −174.5, 0, −386.2, and −237.14 kJ/mol^48^. The standard Gibbs free energy change of the reaction in Eq. 11 can be calculated to be 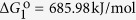, related to a thermodynamic uphill and unfavorable reaction. For the reaction in Eq. 12, it behaves as the oxygen from the gas state to the metal oxide solid state with the release energy equal to the bond energy. Cu-O bond energy of 272.86 kJ/mol is reported^49^. If well-crystalline Cu*Mn*OS has the oxygen bond energy of 272.86 kJ/mol, we assume that the weakened oxygen bond energy in Cu*Mn*OS is 90% of 272.86 kJ/mol or the reaction energy change for one molar 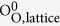 is 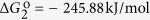. Therefore, the net standard Gibbs free energy of the reaction in Eq. 6 is 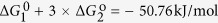, favorable for the reaction in Eq. 6 to occur. From this explanation, the consideration of the chemical potential of lattice oxygen is very important. The schematic diagram for the chemical reaction paths for CO_2_ and H_2_O to form methanol w/o catalyst is shown in Figure 7.

Similar to CO_2_ hydrogenation, the catalytic reaction for aqueous methanol dehydrogenation can be calculated to be 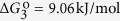 for its net standard free energy change of reaction in Eq. 13, using the standard Gibbs free energies of formation of H_2(g)_, CO_2(g)_, CH_3_OH_(l)_, and H_2_O_(l)_ of 0, −394.38, −166.3, and −237.14 kJ/mole. The gas pressure deviation from the standard state of 1 atm can lead to the free energy change of 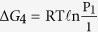 for Eq. 14 and 
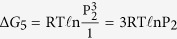
 for Eq. 15. If P_1_ is 0.01 atm for CO_2(g)_ and P_2_ 0.03 atm for H_2(g)_, ΔG_4_ for Eq. 14 is −11.41 kJ/mol and ΔG_5_ for Eq. 15 −26.06 kJ/mol. The net standard Gibbs free energy of the reaction in Eq. 10 is ΔG_3_ + ΔG_4_ + ΔG_5_ = −28.41 kJ/mol, favorable for the reaction in Eq. 10 to occur.


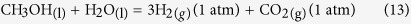



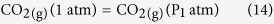



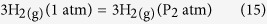


The achievements in CO_2_ hydrogenation and aqueous CH_3_OH dehydrogenation at 25 °C indicate our [Cu^+^]-high and [Cu^+^]-low Cu*Mn*OS catalysts have provided the different interfacial reactions to alter the reaction paths and to obtain fuels without additional inputs of energies and reagents.

The charge transfer between Cu^+^ and Cu^2+^ in semiconductor to provide the electron transport for the n-type or the hole transport for the p-type is understandable. The active lattice oxygen is the key factor for the success of the aqueous CO_2_ reduction and aqueous CH_3_OH dehydrogenation. Basically, ceramic catalysts have long been viewed to be activated at high temperature but cannot at NTP in water. Here our proposed reaction mechanism of Cu*Mn*OS in water for redox reactions at NTP is similar to that of CeO_2_ in water vapor at high temperature with the basis of oxygen vacancy^46^. The thermodynamic calculation also supports the occurrence of the redox reactions. The realization of our CO_2_-CH_3_OH cycle at NTP is strongly related to the reactions between catalyst and water (Equation 1), which are attributed to the low processing temperature for Cu*Mn*OS, the S^6+^ -O bond formation, and the substitution of Mn for Cu to distort the lattice, to weaken the lattice O bonds, and to form the active lattice oxygen. The degraded performance in the H_2_ yield for the 200 °C-annealed Cu*Mn*OS in Table 4 is related to the stronger bonding to deactivate the lattice oxygen for Eq. 1. The photo-excitation result in Table 4 also supports the water oxidation by catalyst as the first reaction step instead of the electron/hole-activated reaction. The weakening of the oxygen bonding to initiate catalytic reactions can be the design strategy for inorganic or heterogeneous catalysts to increase their catalytic activity at mild condition.

**Figure 8:**
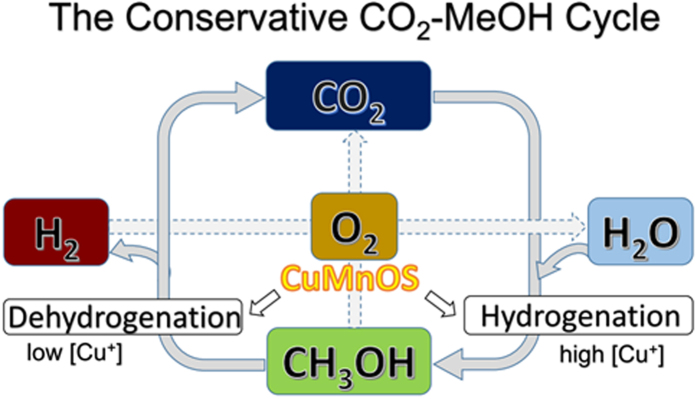


Figure 8 is the schematic illustration to show the conservative CO_2_-CH_3_OH cycle. With the [Cu^+^]-high Cu*Mn*OS catalyst, the greenhouse CO_2_ gas can be recycled and re-utilized by catalytic reduction reaction together with water to form aqueous CH_3_OH solution as the hydrogen liquid carrier, fuel, or the chemical feedstock. With the [Cu^+^]-low Cu*Mn*OS, aqueous CH_3_OH solution can be instantaneously dehydrogenated into H_2_ and CO_2_. Both CH_3_OH and H_2_ are important energy carriers and chemical precursors. The instantaneous H_2_ generation from the CH_3_OH solution can be feasibly applied to the portable appliance, transportation vehicles, power plants etc., after methanol safety has been well considered. The products of CO_2_/H_2_O from the combustion of CH_3_OH/H_2_ can be again recycled and re-utilized. This CO_2_-CH_3_OH cycle occurred at NTP is conservative and renewable.

In summary, we demonstrate the nanoflower-like Cu*Mn*OS catalyst system to complete the conservative CO_2_-CH_3_OH hydrogenation-dehydrogenation cycle in an aqueous solution at normal temperature and pressure without additional energy inputs and reagents. This catalyst system has two different forms. The [Cu^+^]-high Cu*Mn*OS can transport electrons and is used for the aqueous CO_2_ hydrogenation to CH_3_OH at a yield of 21.1 mmol·g^−1^catal.·h^−1^. It is the [Cu^+^]-low Cu*Mn*OS to transport holes and to instantaneously dehydrogenize aqueous CH_3_OH solution into H_2_ at a yield of 7.65 mmol·g^−1^ catal.·h^−1^. In additional to the electron and hole charges, the key factor in completing the CO_2_-CH_3_OH cycle is the active lattice oxygen of Cu*Mn*OS to firstly initiate water oxidation at the catalyst-water interface. The bond weakening concept in forming the active lattice oxygen opens a route to increase the catalytic activity of inorganic catalysts for redox reactions at mild condition. The H_2_ liquid carrier of aqueous CH_3_OH solution with instantaneous H_2_ liberation can provide wide applications in portable appliance, vehicle transportation, power plant etc.

## Additional Information

**How to cite this article**: Chen, X. *et al*. Cu*Mn*OS Nanoflowers with Different Cu^+^/Cu^2+^ Ratios for the CO_2_-to-CH_3_OH and the CH_3_OH-to-H_2_ Redox Reactions. *Sci. Rep.* **7**, 41194; doi: 10.1038/srep41194 (2017).

**Publisher's note:** Springer Nature remains neutral with regard to jurisdictional claims in published maps and institutional affiliations.

## Figures and Tables

**Table 1:** XPS composition analyses of Cu*Mn*OS catalysts.

Catalyst	Molar percentage				Cu molar percentage		Cu^+^/Cu^2+^ molar ratio	O-bonding molar percentage			S molar percentage		S^6+^/S^2-^ molar ratio	O^2^/S^2-^ molar ratio
	Cu	Mn	O	S	Cu^+^	Cu^2+^		O-H	O-Cu^+^ O-Mn	O-Cu^2+^	S^6+^	S^2-^		
Cu*Mn*OS-0	39.6	1.6	21.9	36.9	59.8	40.2	1.49	32.3	33.3	34.4	13.5	86.5	0.156	0.465
Cu*Mn*OS-3	39.0	1.5	21.3	38.2	70.5	29.5	2.39	31.6	35.1	33.3	18.3	81.7	0.224	0.467

**Table 2:** Catalytic reduction of Cr(VI) with Cu*Mn*OS and CuOS catalysts in the dark.

Catalyst	CuOS	Cu*Mn*OS-0	Cu*Mn*OS-1	Cu*Mn*OS-2	Cu*Mn*OS-3	Cu*Mn*OS-4
Reduction content (%)	8.5	57.4	80.3	98.9	100	100

**Table 3:** Cu*Mn*OS catalysts on the reduction of CO_2_ in the dark.

Catalyst	CuOS	Cu*Mn*OS-0	Cu*Mn*OS-1	Cu*M*nOS-2	Cu*Mn*OS-3	Cu*Mn*OS-4
CH_3_OH yield	0.0	7.4	15.9	17.2	21.1	10.4

Note: Unit for the yield: mmol·g^−1^catal.·h^−1^.

**Table 4:** Hydrogen yields over Cu*Mn*OS under different conditions

Condition	Dark/Light	20% methanol + H_2_O	20% ethanol + H_2_O	20% ethanoic acid + H_2_O	H_2_O	ethanol
Catalyst						
CuOS	Dark	0.27	0.23	—	0	0
Cu*Mn*OS-0	Dark	1.54	8.54	2.17	0	0
Cu*Mn*OS-1	Dark	7.65	9.45	—	0	0
Cu*Mn*OS-2	Dark	1.05	2.13	—	—	—
Cu*Mn*OS-3	Dark	2.24	2.03	—	—	—
Cu*Mn*OS-4	Dark	1.65	2.26	—	—	—
Cu*Mn*OS-0	Visible	2.04	2.45	—	—	—
Cu*Mn*OS-0-200	Dark	1.20	—	—	—	—

Note: Unit for the yield: mmol·g^−1^catal.·h^−1^; Cu*Mn*OS-0-200: 200 °C-annealed Cu*Mn*OS.
